# Effectiveness of Mixed Reality in Oral Surgery Training: A Systematic Review

**DOI:** 10.3390/s25133945

**Published:** 2025-06-25

**Authors:** Ruza Bjelovucic, Jan Wolff, Sven Erik Nørholt, Ruben Pauwels, Pankaj Taneja

**Affiliations:** 1 Department of Dentistry and Oral Health, Aarhus University, 8000 Aarhus, Denmark; 2 Department of Maxillofacial Surgery, Head and Neck Cancer Centre, University and University Hospital of Lübeck, 23562 Lübeck, Germany; 3 Department of Radiology, Faculty of Dentistry, Chulalongkorn University, Bangkok 10330, Thailand; 4 Department of Oral Surgery, Sydney Dental Hospital, Sydney, NSW 2010, Australia; 5 School of Dentistry, The University of Sydney, Sydney, NSW 2010, Australia

**Keywords:** virtual reality, augmented reality, simulation training, oral surgery

## Abstract

Background: Advancements in virtual reality (VR) and augmented reality (AR) technologies have the potential to revolutionize surgical training in oral (OS) and maxillofacial surgery (OMFS). This review aims to discuss the current state of VR and AR applications in surgical training, as well as their impact on education and skills acquisition. Methods: Main search terms used in combination: student, education, training, VR, AR, OS, OMFS, oral surgeon, and maxillofacial surgeon. A comprehensive literature search was conducted from 2012 to 2023 using databases including Cochrane Library, Medline, PubMed, Scopus, Embase, Web of Science, and Google Scholar. Results: Out of 545 potential studies, 12 met the inclusion criteria. The review found that VR applications are predominantly used in surgical training, while AR is notably scarce in this context. Conclusions: While VR cannot replace traditional training methods, its integration into surgical education programs could supplement practical experience on phantoms and real patients.

## 1. Introduction

Training in oral and maxillofacial surgery (OMFS) and oral surgery (OS) focuses on developing key skills in diagnosis, surgical planning, and surgical procedures of the head and neck. The average annual intake/graduation of OMFS or OS trainees varies across different regions and institutions. Nonetheless, the World Health Organization (WHO) has estimated that there is a global shortage of 10 million healthcare workers and predicts that this figure will increase to 43 million by 2030 [1]. This directly impacts the ability of universities and hospitals to maintain high standards in healthcare, education, and research.

Traditional training in OMFS relies on trainees learning by observing and practicing on real patients under supervision [2,3]. However, application of the model can be affected by socioeconomic restraints in the operating room [4], patient comfort requirements [5], and restrictions on residents’ working hours [6]. To address these challenges, exploring the integration of technologies such as virtual reality (VR) and augmented reality (AR) alongside the apprenticeship model could address these challenges and potentially offer a more standardized and controlled environment for trainees to develop their surgical skills. In addition, this approach may provide increased interactivity and effectiveness, offering a promising avenue for further investigation regarding its potential benefits in enhancing training experiences [7].

VR is a technology providing users with immersive experience in a computer-generated environment, replicating real or fictional settings, including avatars [8,9]. It provides multi-sensory experiences and enables individuals to interact and manipulate objects in a virtual world, thereby creating a high sense of presence and engagement [10,11]. VR has been sporadically used in healthcare education since 1994 [12]. However, the VR landscape saw limited development until 2012, when the Oculus Rift was released [13] a pioneering VR headset that played a major role in promoting VR technology for entertainment and gaming. Concurrently, the second wave of VR [14] head-mounted displays (HMDs) emerged and were increasingly being used in surgical education, planning, and training [15,16]. Conventional VR and immersive VR (iVR) are two subgroups, with iVR offering a more authentic training experience compared to conventional VR [17]. iVR emphasizes the creation of a realistic and interactive virtual environment for training, incorporating 360° videos, three-dimensional (3D) interaction, and stereoscopic videos [18].

More recently, we have been observing a transition from VR to AR in clinical settings. AR technology enables digitally generated 3D representations to be overlayed onto real-world stimuli [19]. AR can be used with smart phones, tablets, or other devices, providing an immersive experience without requiring dedicated hardware [19]. In contrast, VR always requires HMDs for the same effect. According to systematic reviews conducted by Eckert et al. [20] and Chen et al. [21], it can be observed that the primary application of AR in dentistry lies in the field of surgical procedures and interventions. This is closely followed by the exploration of applications in the fields of therapy and rehabilitation. Despite the growing use of VR and AR across medical disciplines [22,23,24], their specific impact on OMFS and OS training remains understudied. Thus, the objective of the present study is to systematically analyze the available literature focusing on the use of VR and AR in the context of surgical training. This study addresses the following question: do VR or AR tools enhance the development of surgical skills within the field of OMFS and OS? Additionally, the study aims to identify potential areas of future research that may contribute to the translation of these findings into clinical practice, as well as provide an overview of the current state of knowledge.

## 2. Materials and Methods

The systematic review was performed in accordance with the PRISMA guidelines [25] and registered in PROSPERO (ID: CRD42023451623).

A literature search of articles published in English from 2012 (the release year of the Oculus Rift [13]) to 2023 was conducted through the Cochrane Library, Medline, PubMed, Scopus, Embase, Web of Science, and Google Scholar. Any gray literature was retrieved from OpenGrey (https://opengrey.eu/) and WorldCat dissertations (https://www.worldcat.org). A search strategy was developed using a ‘building block searches’ framework and customized for the search databases and their structure. The complete search string is shown in Appendix A. An experienced systematic reviewer (PT) was consulted regarding search terms and keywords. Two reviewers (PT, RB) conducted searches independently on the same day. Duplicates were removed prior to import to the web-based reference program Covidence (www.covidence.org). In Covidence, two reviewers independently screened and identified studies meeting the inclusion criteria. Any disagreement between the reviewers concerning the eligibility of studies was resolved and a third reviewer (JW) reached a consensus.

The inclusion criteria consisted of studies that were published in English which investigated developing surgical skills in undergraduate or postgraduate dental students, dentists, oral surgeons, or maxillofacial surgeons using VR or AR. The exclusion criteria encompassed studies that presented VR/AR simulators as patient case studies, developed VR/AR simulators without testing their efficacy on surgical skills, non-dental studies, non-English language papers, and web-based VR/AR (e.g., studies that lacked HMDs and compatible software). Other types of simulators, such as tablet-based or mobile VR/AR applications, were considered but excluded due to their typically low level of immersion. All the descriptive methodologies, patents, and all the publications in general not identified as “Articles” were discarded. To ensure a rigorous and fair assessment, we used the CASP (Critical Appraisal Skills Program, CASP (Qualitative research), 2018.) checklist, which is a widely recognized tool for appraising the quality of qualitative research [26].

Two reviewers (PT, RB) independently screened half of the titles and abstracts based on the inclusion and exclusion criteria listed above. The inter-rater agreement was assessed to be 91%, and Cohen’s Kappa statistic was 0.80, indicating strong agreement. Therefore, the first author (RB) screened the remaining titles and abstracts and discussed any concerns with the second reviewer (PT).

Given the variability of studies included, we recognized that existing quality assessment tools did not fully address the specific nuances essential to our investigation. Standard risk of bias assessment tools such as ROBINS-I or the Cochrane Risk of Bias Tool were considered during the design of this review. However, these tools are primarily developed for randomized or non-randomized clinical trials and do not sufficiently address the diversity and exploratory nature of the studies included in our review, many of which were observational or pilot studies. Additionally, they do not account for critical VR/AR-specific features such as immersion, interactivity, and haptic feedback. Inspired by the approach taken in the systematic review by [27], which developed a customized quality assessment tool tailored to its subject matter, we chose to create an adapted tool that met the specific needs of this review. This tool was developed by the lead author, in consultation with coauthors, by integrating relevant components from established risk-of-bias domains (e.g., randomization, participant selection, outcome measurement) and extending them with criteria relevant to the context of VR/AR in surgical education. The adapted quality assessment was at the methodological level and consisted of seven key domains related to potential biases: (1) random assignment of participants, (2) biases from study design, (3) participant selection, (4) deviations from intended interventions, (5) biases in outcome measurement, (6) incomplete data, and (7) risks of bias in results. Both reviewers independently conducted the quality assessment. Each domain was rated as having a “low risk,” “unclear risk,” or “high risk” of bias to ensure a transparent and comprehensive evaluation of the included studies.

## 3. Results

The results are presented in the following order: the study selection process, quality assessment of the included studies, an overview of study characteristics, and a synthesis of findings related to the application of VR and iVR in OS and OMFS.

### 3.1. Study Selection

**Figure 1:**
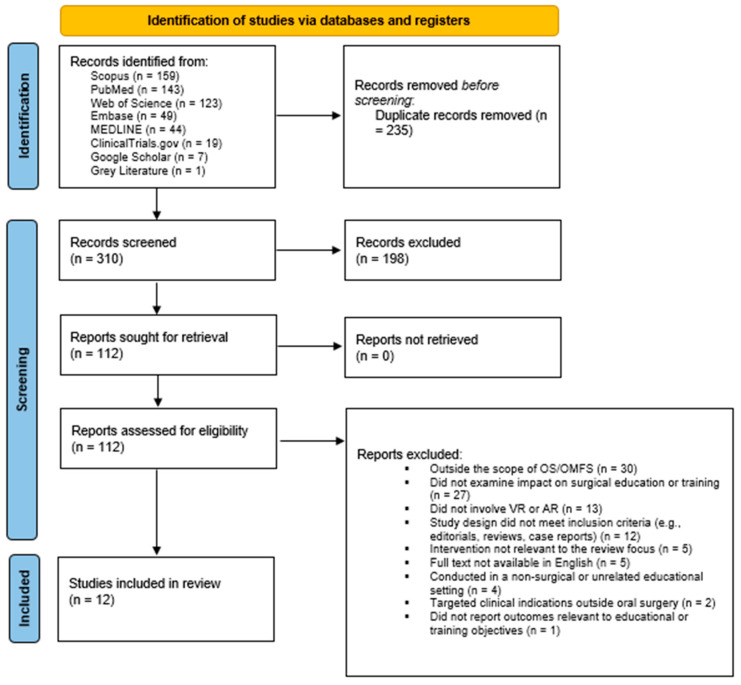
PRISMA flowchart of the study selection process.

A total of 545 potential studies were retrieved from seven databases. After eliminating 235 duplicates, 310 articles were assessed based on their title and abstract, of which 198 were removed as they did not meet the inclusion criteria. Thus, 112 full texts were downloaded and evaluated for eligibility, resulting in the exclusion of 101 studies. Consequently, 11 studies were eligible to be included for the present study. After screening grey literature, one study met the criteria and was added to the list, resulting in a total of twelve studies (Figure 1).

### 3.2. Quality Assessment

**Figure 2:**
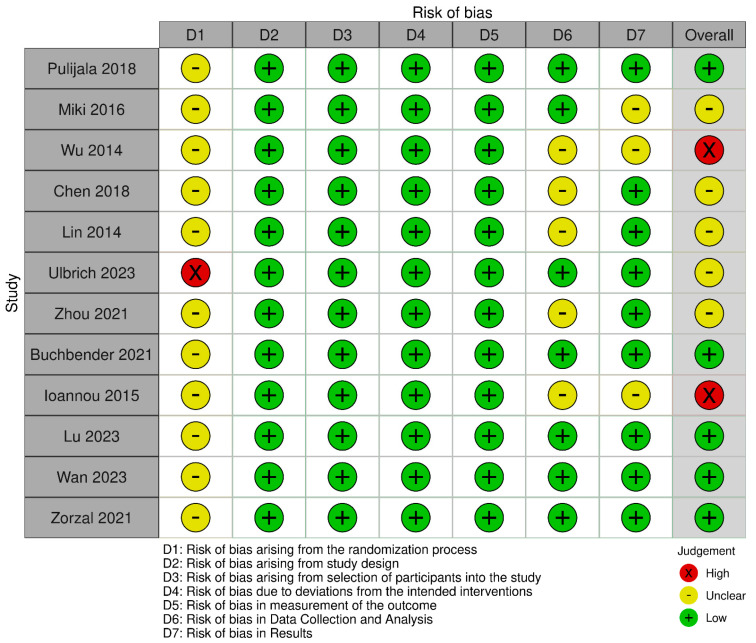
Risk of bias analysis of each included study.

**Figure 3:**
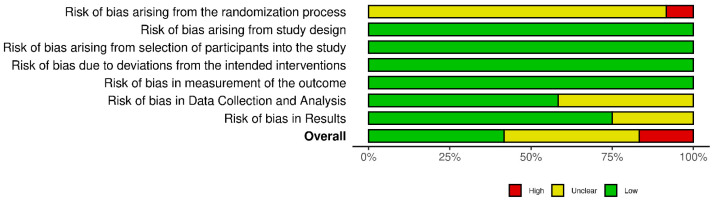
Overall risk of bias analysis.

Five studies [15,17,28,29,30] achieved a satisfactory score regarding the quality assessment (Figure 2 and Figure 3). The remaining studies (N = 7, where N = number of studies) [31,32,33,34,35,36,37] were identified to have a degree of potential bias in terms of data collection, analysis, and/or results. The quality assessment score did not affect inclusion, and no studies were excluded based on their rating. Notably, no studies randomly assigned participants.

### 3.3. Study Characteristics

The systematic review comprised of studies from China (6), Germany (2), Japan (1), United Kingdom (1), Australia (1), and Portugal (1). Sample sizes varied between seven and fifty-nine participants, with the majority being oral and maxillofacial surgeons (N = 5) [28,29,32,33,35], dental students (N = 3) [15,32,36] or medical students (N = 1) [33], and dentists (N = 2) [15,30]. Nine [15,17,30,31,32,33,34,35,36,37] of the twelve studies compared VR scenarios, while the others utilized iVR technology [17,28,29]. The characteristics of the studies, intervention details, outcomes, and application are presented in Table 1. VR and iVR were primarily used for acquiring skills in orthognathic surgery (N = 4) [17,28,33,34], followed by implantology (N = 3) [30,31,37]. Other studies explored the potential of VR or iVR for virtual surgery planning (VSP) [32], bone removal [36], apicectomy and wisdom tooth removal [15], removal of the submandibular gland [35], and craniomaxillofacial trauma treatment [29]. However, there was no evidence found of AR being utilized for developing OMFS and OS skills in this systematic review. Therefore, the results and Table 1 do not include AR applications.

### 3.4. VR Simulators

To better understand how VR has been applied in surgical training, the studies are categorized based on their clinical focus. The following subsections highlight VR-based simulation tools used in specific areas, beginning with orthognathic surgery, one of the most common applications.

#### 3.4.1. VR Simulators in Orthognathic Surgery

Two studies [33,34] specifically focused on the bone-sawing procedure through the maxilla. Lin et al. [33] demonstrated that VR-based training enables accurate transfer of osteotomy skills to real-world tasks on skull models. Their study also showed that VR training significantly reduced bone removal time between the first and last trials. Similarly, another study [34] validated successful skill transfer using VR, employing the Omega.6 (Force Dimension, Nyon, Switzerland) simulator with a 3D immersive workbench and high-resolution display. Feedback from both novice trainees and experienced surgeons confirmed the system as a practical tool for mastering OMFS procedures.

##### VR Simulators in Implantology

Zhou et al. [31] created a VR simulation for implant surgery in the mandible, showing that Group A (trained with VR) achieved better accuracy in mesiodistal, buccal-lingual dimensions, depth, and angle compared to Group B (trained traditionally). The VR group demonstrated higher success rates, with participants gaining confidence in the system’s usability and intuitiveness. Another study [30] confirmed that VR facilitates effective learning for implant placement, emphasizing its user-friendly and accessible nature.

##### VR Simulators in VSP

A study on virtual surgical planning (VSP) [32] compared a VR environment with a desktop screen (DS) for training. The VR environment demonstrated clear advantages, including a shorter learning curve and nearly double the segmentation speed, enabling faster achievement of high-quality outcomes. Participants also reported less fatigue while working in VR compared to DS.

##### Other VR Simulators in OFMS and OS

Miki et al. [35] also developed a VR training system for inexperienced oral surgeons to practice submandibular gland removal. After training, surgeons showed a significant reduction in surgical time and errors, achieving error-free performance after ten sessions. Conversely, Buchbender et al. [15] found that the Kobra VR surgical simulator was not significantly preferred over traditional plastic model training. However, it was regarded as a complementary tool that could benefit both students and educators. Ioannou et al. [36] further reported that VR training for bone removal improves trainees’ movement, optimizing efficiency and task execution without compromising outcomes.

#### 3.4.2. iVR Simulators

In contrast to traditional VR, immersive virtual reality (iVR) provides greater user engagement through full 3D environments and motion tracking. The studies involving iVR are grouped below by their clinical application.

##### iVR Simulators in Orthognathic Surgery

Two studies explored the use of iVR as a training tool for orthognathic surgical education [17,28]. Pulijala et al. [28] conducted a study using the Oculus Rift Development Kit 2 VR HDMs (Reality Labs, Menlo Park, CA, USA) and Leap Motion controllers (Ultraleap, Bristol, UK) to a provide realistic experience of the Le Fort I osteotomy. Experts rated the iVR scenario as highly relevant to the current curriculum, giving it an average score of 4.53 out of 5. Wan et al. [17] utilized the HTC VIVE Pro 2 (HTC Corporation, Taoyuan City, Taiwan) to perform bimaxillary orthognathic surgery on a virtual patient. Their findings demonstrated that iVR was both realistic and beneficial for enhancing surgical skill acquisition.

##### iVR Simulator in Trauma Treatment

Recently, 25 maxillofacial surgeons evaluated an iVR training tool for pre-hospital craniomaxillofacial trauma treatment [29]. The tool emphasized interactivity in a specific battlefield scenario and demonstrated a satisfactory level of face and content validity. This tool could be used effectively by novices to improve the detection of cerebrospinal fluid and enhance understanding of procedures.

## 4. Discussion

Technological innovations, such as integration of computer-assisted navigation systems [38,39] and artificial intelligence [40,41], have enhanced the fields of OMFS and OS. The use of VR in surgical training has been successfully demonstrated in other medical specialties, such as laparoscopic surgery, where it has been shown to offer comparable training outcomes to traditional training [42]. Integrating VR into OMFS and OS training enhances surgical skill acquisition and anatomical knowledge, particularly benefiting undergraduate students and those in their specialization phase. In summary, all 12 articles collectively highlight the transformative impact of VR on dental surgical training. VR technologies not only improve the realism of simulated surgical environments but also refine the skills of surgical trainees. These results highlight the academic and practical capacity of VR as an invaluable asset for improving surgical training and developing expertise. However, at present, AR is still absent from the teaching landscape of OMFS and OS.

### 4.1. VR and OMFS Skills

After the studies were assessed for quality, the results showed that some of the papers considered VR as an additional tool [15,17,28,31,37] for learning. This is due to VR addressing specific challenges and exploit unique characteristics that may not be fully captured by traditional educational methods. Its immersive nature provides a realistic environment for learners to practice [35] and refine surgical skills in a risk-free environment. In addition, VR facilitates scenario-based learning, allowing learners to experience different clinical situations and refine decision-making skills [29,35].

In an academic context, VR aligns with pedagogical principles and enhances both cognitive and psychomotor aspects of learning [43,44]. VR was recognized for its environmental friendliness and its ability to engage students in impactful learning experiences [30,31,34]. VR has emerged as a key tool in shortening the learning curve and, therefore, providing a faster and more efficient educational experience [28,29,32,34,35]. VR’s accelerated learning curve can be attributed to several key factors. VR creates an interactive learning environment that significantly increases engagement and motivation, resulting in improved information retention and comprehension [45,46]. In addition, VR allows learners to manipulate objects and navigate environments in ways that are not possible in traditional learning [32,47]. This feature facilitates a deeper understanding of complex subject matters. Moreover, VR provides a safe and controlled environment where learners can practice and make mistakes without real-world consequences. In essence, the combination of immersion, interactivity [28,29], and experiential learning inherent in VR underlines its key role in shortening and accelerating the learning curve.

To date, VR has mainly been developed for training in orthognathic surgery, such as Le Fort I osteotomy, which involves manipulating delicate anatomical structures and precise surgical maneuvers. Furthermore, a study [32] has demonstrated that VR can aid in planning. However, the removal of wisdom teeth, a common procedure in dentoalveolar surgery, seems to be undervalued in VR applications, despite requiring skill and precision. The complexity and risk factors associated with wisdom tooth extraction are often lower compared to procedures such as Le Fort I osteotomy [48,49]. Therefore, the use and investment of VR technology for wisdom tooth extraction may not be as widespread or necessary. However, it could still be beneficial in training students, particularly in cases involving complications or challenging anatomical positions. A notable advantage of VR is its timesaving quality and adaptability to individual student needs [34]. Most VR programs can analyze success and errors, allowing for more detailed feedback discussions between teacher and student. Buchbender et al. [15] identified that students develop greater self-determination and self-motivation in the VR. Miki et al. [35] and Chen et al. [37] explored VR simulators in dental surgery, demonstrating its potential to assist inexperienced students in acquiring a tactile sense of force and thereby improving novice skill levels. This advancement sparks optimism for the next decade, anticipating a generation of novices who may benefit from such technology, in turn allowing them to more confidently undertake advanced surgical procedures.

### 4.2. Technical Limitations of VR

The results collected in this systematic review indicate that there are still limitations and challenges that need to be addressed, such as poor quality of graphics and visuals, because these can diminish the overall experience [17,28,32]. Pulijala et al. [28], in their validation of VR in Le Fort I osteotomies, struggled with an effect called the “screen door effect” (SDE). This is a perceptual artifact occurring when the visibility of inter-pixel spaces inherent to certain pixel grid arrangements on display technologies like OLED or LCD screens create a grid-like pattern [50]. Put simply, the image appears as if it is behind a screen door. Pixel shifting is often used to reduce the visibility of the grid-like pattern created by the gaps between pixels to mitigate the SDE. By slightly shifting the position of pixels to cover or minimize these gaps, a smoother and more continuous image is perceived [50]. Furthermore, one of the main complaints among participants in VR and iVR studies is dizziness [17,28,31]. Dizziness is one of the VR-induced symptoms of cybersickness (CS). Empirical evidence suggests that a significant proportion of people, ranging from 60 to 95%, experience varying degrees of CS when engaging with a virtual environment [51]. CS typically appears 10–15 min after a user is immersed in a VR environment and disappears again 10–15 min after the user exits [52]. The etiology of CS is unknown, but three theories have been proposed. The sensory conflict theory is based on a discrepancy between visual, vestibular, and proprioceptive senses. The postural instability theory describes a psychologically perceived inability to maintain equilibrium, leading to similar effects to motion sickness. Lastly, the eye movement theory suggests that visual stimuli in virtual environments can affect the eyes. It seems that the type of movement is a primary factor in CS. CS not only affects the quality of VR experiences but also has the potential to disrupt cognitive and motor functions. This is particularly problematic in domains such as education, clinical applications, and training, where these skills are critical for learning. Dahlman et al. [53] posits that motion sickness may severely impair verbal working memory. Supporting this, Kourtesis et al. [54] found that CS negatively influenced visuospatial working memory and psychomotor skills. Their study, involving 30 participants aged 20–45, examined the predictors of CS and its impact on cognitive and motor performance. Participants completed a rollercoaster VR experience, with assessments conducted before, during, and after the session. The results identified motion sickness susceptibility and gaming experience as significant predictors of cybersickness, with the latter leading to more resilience. However, in their meta-analysis, Caserman et al. [51] found that the latest generation of HMD devices have significantly fewer problems in terms of CS. To prevent CS during surgery simulations in VR and prolong enjoyment, it is advisable to provide room-scale environments where users can walk naturally. Adjustments such as slower motion, simplified visual effects, and shorter session durations can be implemented to accommodate CS [55]. Another potential solution is to create a more enjoyable VR experience by tailoring it to each person’s sensitivity to motion sickness and sensory limits. Adapting VR content in real time with the help of machine learning algorithms could help minimize discomfort for users [56].

Another limitation is that some of the studies did not include haptic feedback [17,28,29,30,32,37]. Haptic feedback is broadly divided into two categories: kinesthetic feedback, which involves force sensations, and cutaneous feedback, which pertains to tactile sensations [57,58]. Kinesthetic feedback is essential for developing the motor skills required to handle surgical tools with precision. In tandem, the tactile feedback system offered precise real-time responses to gripping forces, thereby ensuring accurate tool–tissue interactions and reducing the risk of tissue damage in simulated environments.

It is believed that haptic feedback in minimally invasive surgery may reduce errors and improve training in VR [59]. However, reproducing the mechanical properties during bone cutting, milling, and drilling is challenging due to various influencing factors, such as vibration frequency, bone density, and surgical instrument feed and speed. Constructing a mathematical model for simulating forces on bony tissue is a complex task due to the intricate nature of skeletal structures. A dental implant surgery simulator, based on the haptic device Omega.6 (Force Dimension, Nyon, Switzerland) and Computer Haptics and Active Interface (CHAI3D), has been introduced in prior work [37]. The simulator allows the diameter and speed of the drill to be varied. Wu et al. [34] replicated bone operation values using same haptic feedback device, a 3D immersive workbench, and a 2D LCD monitor. Force measurements were taken on eight cadaver mandibles to record force values and directions during surgery. Regression equations were established to determine the relationship between these parameters and haptic force. However, the maximum force of the simulator is insufficient for real-world operations. Tactile feedback aids surgeons in identifying and navigating around structures, reducing the risk of inadvertent injury, and improving the overall safety of the procedure. Haptic feedback could also improve the experience of VR craniomaxillofacial trauma treatment [29]. While haptic simulators offer significant advantages in OMFS/OS applications, they are currently underutilized, possibly due to a lack of scientific studies documented in the literature [60]. Addressing this gap, a primary future challenge lies in the delivery of accurate real-time haptic sensations during simulations. A notable advancement in this area is the incorporation of advanced haptic feedback into robotic surgical training systems. One such innovation is a robotic endotrainer [61] designed in a master–slave configuration similar to the DaVinci surgical robot (Intuitive Surgical, Sunnyvale, CA, USA). This system was developed as a cost-effective training tool for robotic surgery, integrating both kinesthetic and tactile feedback mechanisms to enhance realism and improve the overall training experience. These advancements highlight the transformative potential of haptic feedback in surgical education and its capacity to elevate the effectiveness of training simulations.

### 4.3. Acquisition of Surgical Skill Limitation

Most participants mentioned that there was a lack of soft tissue, incision, flapping, and bleeding in VR simulations [15,30,31,34,37]. Soft tissue in OMFS/OS is a critical consideration for successful outcomes. In addition to orthognathic surgery, advancements in VR simulations have shown promising results in training programs for OMFS procedures. Soft tissue in VR OMFS is created using a variety of methods and models. One approach is to compute soft tissue characteristics using trade-offs between real-time capability and deformation accuracy [62]. Another approach is to use a large number of spheres to represent tissues in the VR simulation; however, to improve bleeding and soft tissue, Miki et al. suggested that three-dimensional structures of tissues should be created from the outset [35]. This means that the VR models should accurately represent the anatomical structures and surrounding tissues in a 3D format. Lastly, a lack of communication was identified in one study [15]. Effective communication between the surgical team is essential during surgery to ensure patient safety and successful outcomes. In the context of VR surgical training, the immersive and interactive nature of the experience provides a unique opportunity to simulate real-life surgical scenarios. Further incorporating aspects of communication and collaboration into VR surgical training could enhance the overall experience for trainees. This could include simulating scenarios where trainees need to communicate with other team members or patients, make critical decisions, and coordinate actions effectively.

### 4.4. Augmented Reality

In this study, we did not identify any research involving applications of AR using HDMs for teaching or acquiring surgical skills. Nonetheless, it is important to highlight the current state of AR and its applications in OMFS/OS surgical practice. For example, one study demonstrated the use of Microsoft HoloLens in three common oral surgery procedures, concluding that AR is a valuable tool for visualizing and manipulating images to assist in surgical approaches and enhance patient communication [63]. Another noteworthy study showcased the use of AR in the assisted surgical removal of mandibular odontogenic cysts [64]. These examples underscore the potential of AR as a complementary tool to other computer-assisted technologies.

In conclusion, the application of AR in OMFS/OS education and skill development holds significant promise. Furthermore, AR’s integration into live surgeries offers the potential for trainees to observe complex procedures in detail, guided by augmented overlays of anatomical structures and surgical plans. By combining these capabilities with advancements in high-immersion AR technologies, the future of OMFS/OS training could shift toward a more interactive, effective, and accessible learning approach.

### 4.5. Future Work

The advancement of VR is deeply connected to progress in technology. VR has now entered what is often referred to as the “second wave,” and its full potential can be unlocked through improvements in human–computer interaction (HCI). HCI refers to interactive systems designed to enable seamless and natural communication between humans and machines [65,66]. Among these technologies is haptic feedback, which has been discussed earlier in this article as a key component of enhancing VR experiences.

The integration of machine learning and artificial intelligence is poised to play a critical role in the adoption and expansion of VR in medicine and beyond [65]. Machine learning algorithms can support tasks such as recommendation systems, predictive modeling, user profiling, and behavior analysis. By leveraging AI, computers can predict user needs, automate repetitive processes, and provide intelligent assistance, ultimately enriching the overall user experience.

Another important trend in HCI is the development of gesture recognition and natural user interfaces. These systems allow users to interact with technology through intuitive gestures and movements, offering a more natural way to engage with devices [67]. By utilizing sensors and machine learning algorithms, gesture recognition systems can interpret hand motions, body language, and facial expressions, enabling smoother and more expressive interactions. By combining these cutting-edge technologies, VR in OMFS/OS holds the potential to redefine development of skills, optimize procedural accuracy, and ultimately improve patient care, marking a significant step forward in the field.

### 4.6. Overall Impact

This comprehensive review of the role of VR in OMFS and OS underscores its potential as an adjunct to the development of surgical skills. VR has demonstrated the ability to improve skills, accelerate the learning curve [28,29,32,34,35], and promote familiarity with surgical anatomical structures [28,35,37]. Despite efforts to comprehensively review the literature, this study encountered limitations inherent in the predominance of non-experimental study designs, potential biases during quality assessment, and the absence of randomized control trials. A notable concern in the current state of the field is the lack of randomized control trials (RCTs). This methodological gap raises questions about the robustness of the existing evidence base and emphasizes the preliminary nature of the evidence so far. Future efforts should focus on addressing this gap by prioritizing the implementation of rigorous RCTs. This strategic shift will not only strengthen the empirical foundation of VR applications in OMFS/OS but also increase the reliability and applicability of the findings. Moreover, it is essential to explore the complementary role of AR to further enrich the educational landscape in OMFS/OS. This broader perspective ensures a comprehensive examination of these technologies in the field of OMFS/OS in the broadest context possible. Future research directions may also be highlighted.

## 5. Conclusions

In conclusion, this study aimed to investigate whether VR or AR tools enhance the development of surgical skills within the field of OMFS and OS. The findings indicate that VR shows promise in bridging the gap between theoretical knowledge and practical skills. However, it cannot replace the need for practice on phantoms and real patients at this stage. Although AR technology has potential, it has not yet been widely adopted in surgical training within the scope of OMFS and OS. While our synthesis provides insights into the current state of VR, it is crucial to acknowledge the need for further research, particularly through experimental studies and RCTs. Future research should also explore the long-term impact of VR surgical training on the performance and outcomes of trainees, as well as the potential cost-effectiveness of implementing VR training programs in surgical education.

## Figures and Tables

**Table 1:** Overview of selected studies.

Author, Year of Publication, and Location	Aim	Hardware	Software	Primary Findings	Outcome Metrics	Design of Study	Sample Size	Type of Technology Used	iVR/VR Validation	Surgical Scenario
Buchbender et al. [15] (2021) Germany	Evaluated the use of an oral surgery simulator in dental education	Kobra VR simulator system (Haptikfabriken AB, Stockholm, Sweden)	Not applicable	Simulator provides controlled environment for surgical practice, improving practical skills. Simulation measures objective parameters (bone, tooth substance, soft tissue removal) with no significant differences found between student and dentist groups	Bone removed (mm^3^) Infected tissue removed Guttapercha removed Enamel removed Dentin removed Pulp removed Procedure time	Case-control study	59 (49 students and 10 dentists)	VR	VR is an additional method to conventional surgery training using plastic models	Apicoectomy Wisdom Tooth Extraction
Chen et al. [37] (2018) China	Develop a haptic simulator for dental implant surgery to improve trainees’ drilling performance	Omega.6 force-feedback device (Force Dimension, Nyon, Switzerland), Display 300 (SenseGraphics, Göteborg, Sweden), and 3D glasses	IDE and Toolkits: Visual Studio 2010 (Microsoft Corporation, Redmond, WA, USA), Eigen, OpenSceneGraph, CHAI3D (Force Dimension, Nyon, Switzerland). Imaging/Visualization: Mimics (Materialise NV, Leuven, Belgium), 3dMD stereo (3dMD LLC, Atlanta, GA, USA), AABB (AABB, Inc., Seoul, South Korea) and Qt (Qt Group Plc, Espoo, Finland) /VTK (Kitware, Inc., Clifton Park, NY, USA) frameworks	Integrated haptic-based and visual simulator aimed to enhance surgeons’ skills without cadaver experiments Novice surgeons positively evaluated simulator usability, visual authenticity, and haptic authenticity	Incision/cutting force–depth and force–time curves Real-time performance metrics (e.g., latency, stability)	Pilot study	30	VR	VR provides an alternative training method for surgeons to enhance their dental implant surgical skills and experiences	Implantology
Ioannou et al. [36] (2015) Australia	Assess VR training effects on dentistry students learning a new oral surgery task	VR simulator and Phantom 1.5 High Force haptic device (Sensable Technologies/3D Systems, Rock Hill, SC, USA)	Ascension TrakSTAR (NDI—Northern Digital Inc., Waterloo, ON, Canada)	The simulator technique is similar, but not identical, to real-world technique Simulator practice showed a statistically significant decrease in the total path length compared to the control group	Total time Drilling time Number of burr lifts Total strokes Mean stroke duration Mean stroke distance Mean stroke speed % straight strokes % round strokes Drilling path length Total path length Voxel overlap with expert (similarity) Tooth damage (voxel-based) Unnecessary bone removed	Pilot study	14 dental students	VR	VR simulator training improved trainees’ motion economy without affecting task outcome	Bone removal
Lin et al. [33] (2013) China	Evaluate the effects of a surgical training simulator with haptic feedback	Same as Chen et al. [37]	Same as Chen et al. [37]	Novices benefited more from VR showing improvements in safe force learning, stable hand control, and overall performance compared to experienced surgeons VR simulator demonstrated transfer validity to real sawing operations	Force–time curves Force–depth curves during insertion and cutting	Experimental study	25 (9 OFMS surgeons and 16 novices)	VR	VR could be used as a training alternative for trainees in bone-sawing	Le Fort I osteotomy
Lu et al. [29] (2023) China	Validation of iVR training tool for pre-hospital craniomaxillofacial trauma treatment	HTC Vive Pro 2 (HTC Corporation, Taoyuan City, Taiwan) and controllers	Unity Engine (Unity Technologies, San Francisco, CA, USA) Obi Fluid Plugin VRTK (Virtual Reality Toolkit Extend Reality Ltd, Birmingham, UK)	Surgeons found the VR training tool’s content, appearance, realism, and anatomy accurate They agreed that it is a valuable addition to the curriculum	Task completion (e.g., ventilation, hemostasis, CSF detection, fracture fixation)	Experimental study	25 surgeons	iVR	VR was positively rated by surgeons, indicating agreement with its validity and applicability	Craniomaxillofacial trauma treatment
Miki et al. [35] (2016) Japan	Create a VR training system for endoscope-assisted submandibular gland removal	Two Geomagic Touch (Geomagic Technologies/3D Systems, Rock Hill, SC, USA) haptic devices	Not applicable	VR resulted in shorter surgery durations and improved performance in endoscope-assisted surgery The content and expression of the system are insufficient, and improvements are needed in the range of operations and visual effects of bleeding	Motion analysis-derived performance metrics comparing VR simulator vs. ovine jaw model	Descriptive study	10 oral surgeons	VR	VR is a valuable tool in developing surgical skills	Removal of the submandibular gland
Pulijala et al. [28] (2018) UK	Test validity and usefulness of iVR for surgical training	Oculus Rift Development Kit 2 (DK2) VR headset (Reality Labs, Menlo Park, CA, USA) and a Leap Motion controller (Ultraleap, Bristol, UK), 6 GoPro Hero (GoPro, San Mateo, CA, USA) video cameras	Not applicable	iVR offers comprehensive training with interactive 3D anatomy and instruments Surgeons suggest adding haptic feedback	Self-confidence scores (5-point Likert scale)	Experimental study	9 surgeons	iVR	iVR is a valid training tool	Le Fort I osteotomy
Ulbrich et al. [32] (2023) Germany	Evaluate VR advantages for segmentation	Vive Pro (HTC Corporation, Taoyuan City, Taiwan), Controller 2.0 (Valve Corporation, Bellevue, WA, USA)	IPS CaseDesigner (KLS Martin Group, Tuttlingen, Germany)	Participants perceived the VR environment as more intuitive and less exhausting VR showed a faster learning curve and nearly doubled segmentation speed compared to a desktop screen	Self-reported confidence (pre- and post-course questionnaires) Subjective evaluation of software usability and course relevance (Likert scale)	Crossover study	6 (5 OMFS + 1 dentistry student)	VR	VR for surgical planning offers faster learning and increased work speed compared to 2D, making it preferable for users and enhancing its integration into clinical practice	VSP planning
Wan et al. [17] (2022) China	Assess the validity of an iVR training system for orthognathic surgery	HTC Vive Pro 2 (HTC Corporation, Taoyuan City, Taiwan)	Unreal Engine 4 (Epic Games Inc., Cary, NC, USA)	Participants strongly agreed on the system’s fidelity, virtual environment, instruments, anatomy, and surgical procedures They believed immersive iVR was essential for surgical education	Procedural duration Number of instrument selection errors Instrument position and angular errors Number of prompts required to proceed	Pilot experimental study	14	iVR	VR could supplement or potentially replace traditional surgical training methods	Double jaw orthognathic surgery
Wu et al. [34] (2013) China	Develop a virtual training system for OMFS	3D immersive workbench (Display 300, SenseGraphics, Göteborg, Sweden) and a force-feedback haptic device (Omega.6, Force Dimension, Nyon, Switzerland)	Not applicable	Virtual training system proved efficient and cost-effective for novice training VR system received positive feedback, indicating its potential for skill learning in OMFS	Subjective user feedback	Pilot study	25	VR	VR provides a realistic and immersive training environment	Le-Fort I osteotomy
Zhou et al. [31] (2021) China	Evaluate application of VR in an implant training system	HTC Vive helmet and handle (HTC Corporation, Taoyuan City, Taiwan)	Mimics 17.0 (Materialise NV, Leuven, Belgium)	VR demonstrated higher scores in postoperative assessment, indicating a better grasp of the simulator compared to traditional training methods VR training group scored significantly higher in anatomical mastery, surgical vision, cavity preparation, implant placement sense, and process mastery compared to conventional training group.	Implant deviation (depth, angle), post-training scores	Observational study	30	VR	VR is an appropriate alternative to 2D conventional simulations methods	Implantology
Zorzal et al. [30] (2020) Portugal	Evaluate VR usability and acceptance in training sessions	Oculus Rift (Reality Labs, Menlo Park, CA, USA) and a Leap Motion controller (Ultraleap, Bristol, UK)	Not applicable	Versatile and portable tool for learning implant placement VR for dental implant placement showed good usability and high learnability VR is considered user friendly	User acceptance, usability	Exploratory study	16 dentists	VR	VR system helps users evaluate their work and identify areas to improve their skills	Implantology

## Data Availability

Not applicable.

## Appendix A. Search Details

**Database**	**Search Details**	**Results**
PubMed	((student*) OR (education) OR training) AND (((((virtual reality[tiab]) OR (“virtual reality”[MeSH Terms])) OR ((augmented reality[tiab]) OR (“augmented reality”[MeSH Terms]))) OR (((((((“Haptic Technology”[Mesh]) OR (Simodont[tiab])) OR (virtual simulation[tiab])) OR (Virtual Surgical Planning[tiab])) OR (Haptic[tiab])) OR (simulator[tiab])) OR (“Simulation Training”[Mesh]))) AND ((((“Surgery, Oral”[Mesh]) OR (“Oral Surgical Procedures”[Mesh])) OR (“Oral and Maxillofacial Surgeons”[Mesh])) OR ((((((oral surgery[tiab]) OR (Maxillofacial Surgery[tiab])) OR (Maxillofacial Surgeon[tiab])) OR (Maxillofacial Surgeons[tiab])) OR (oral Surgeons[tiab])) OR (oral Surgeon[tiab])))) Filters: from 2012–2023	141
Web of Science	((TS = (student*)) OR TS = (education)) OR TS = (training) AND ((TS = (virtual reality)) OR TS = (augmented reality)) OR TS = (simodont)) OR TS = (virtual simulation )) OR TS = (Virtual Surgical Planning )) OR TS = (haptic)) OR TS = (simulator ) AND ((TS = (oral surgery)) OR TS = (Maxillofacial Surgery)) OR TS = (oral surgeon)) OR TS = (oral surgeons)) OR TS = (maxillofacial surgeon)) OR TS = (Maxillofacial Surgeons) Filters: from 2012–2023	123
Scopus	Advanced query ( ( TITLE-ABS-KEY ( maxillofacial AND surgeons ) ) OR ( TITLE-ABS-KEY ( maxillofacial AND surgeon ) ) OR ( TITLE-ABS-KEY ( oral AND surgeon ) ) OR ( TITLE-ABS-KEY ( oral AND surgeons ) ) OR ( TITLE-ABS-KEY ( maxillofacial AND surgery ) ) OR ( TITLE-ABS-KEY ( oral AND surgery ) ) OR ( TITLE-ABS-KEY ( oral AND surgery ) ) ) AND ( ( TITLE-ABS-KEY ( student* ) ) OR ( TITLE-ABS-KEY ( education ) ) OR ( TITLE-ABS-KEY ( training ) ) ) AND ( ( TITLE-ABS-KEY ( virtual AND reality ) ) OR ( TITLE-ABS-KEY ( virtual AND reality ) ) OR ( TITLE-ABS-KEY ( augmented AND reality ) ) OR ( TITLE-ABS-KEY ( simodont ) ) OR ( TITLE-ABS-KEY ( virtual AND simulation ) ) OR ( TITLE-ABS-KEY ( virtual AND surgical AND planning ) ) OR ( TITLE-ABS-KEY ( haptic ) ) OR ( TITLE-ABS-KEY ( simulator ) ) ) AND PUBYEAR > 2011 AND PUBYEAR < 2024	159
MEDLINE via Ovid	#1education or student* or training).mp. or Simulation Training/ #2 virtual reality or augmented reality or simodont or virtual simulation or virtual surgical planning or haptic or simulator).mp. or Haptic Technology/or Virtual Reality/or Augmented Reality/ #3 oral surgery or oral surgeon or maxillofacial surgeon or maxillofacial surgery).mp. or Surgery, Oral/or “Oral and Maxillofacial Surgeons”/ #4 #1 AND #2 AND #3 Applied filters: 2012–2023	44
EMBASE	(education:ab,ti OR student:ab,ti OR ‘medical education’:ab,ti) AND (‘virtual reality’:ab,ti OR ‘augmented reality’:ab,ti OR simodont:ab,ti OR ‘simulation’:ab,ti OR ‘virtual surgical planning’:ab,ti OR simulator:ab,ti OR haptic:ab,ti) AND (‘oral surgery’:ab,ti OR ‘dental surgeon’:ab,ti OR ‘maxillofacial surgery’:ab,ti), Filters: 2012–2023	51
Google Scholar	allintitle: education OR student OR training AND “virtual reality” OR “augmented reality” OR “simodont” OR “virtual simulation” OR “virtual surgical planning” OR “haptic” OR “simulator” AND “oral surgery” OR “maxillofacial surgery”	8
Cochrane Central Register of Controlled Trials	#1 (education):ti,ab,kw OR (student):ti,ab,kw OR (“training”):ti,ab,kw (Word variations have been searched) #3 #1 AND #2 #4 (“maxillofacial surgery”):ti,ab,kw OR (maxillofacial NEXT (surgeon OR surgeons)):ti,ab,kw OR (“oral surgery”):ti,ab,kw OR (“oral surgeon”):ti,ab,kw OR (oral surgeons):ti,ab,kw (Word variations have been searched) #5 MeSH descriptor: [Surgery, Oral] explode all trees #6 MeSH descriptor: [Oral and Maxillofacial Surgeons] explode all trees #7 #4 OR #5 OR #6 #8 #1 AND #7 #9 (“virtual reality”):ti,ab,kw OR (augmented reality):ti,ab,kw OR (virtual simulation OR simodont OR virtual surgical planning):ti,ab,kw OR (“haptic”):ti,ab,kw OR (“simulator”):ti,ab,kw (Word variations have been searched) #10 MeSH descriptor: [Virtual Reality] explode all trees #11 MeSH descriptor: [Augmented Reality] explode all trees #12 #9 OR #10 OR #11 #13 #8 AND #12	19
